# False-positive recalls in the prospective Malmö Breast Tomosynthesis Screening Trial

**DOI:** 10.1007/s00330-023-09705-x

**Published:** 2023-05-05

**Authors:** Kristin Johnson, Jakob Olinder, Aldana Rosso, Ingvar Andersson, Kristina Lång, Sophia Zackrisson

**Affiliations:** 1 Department of Translational Medicine, Radiology Diagnostics, Lund University, Malmö, Sweden; 2 Department of Medical Imaging and Physiology, Skåne University Hospital, Malmö, Sweden; 3 Department of Clinical Sciences, Geriatric Medicine, Lund University, Skåne University Hospital, Malmö, Sweden; 4 Unilabs Mammography Unit, Skåne University Hospital, Malmö, Sweden

**Keywords:** Female, Mammography, Breast neoplasm, Mass screening

## Abstract

**Objectives:**

To evaluate the total number of false-positive recalls, including radiographic appearances and false-positive biopsies, in the Malmö Breast Tomosynthesis Screening Trial (MBTST).

**Methods:**

The prospective, population-based MBTST, with 14,848 participating women, was designed to compare one-view digital breast tomosynthesis (DBT) to two-view digital mammography (DM) in breast cancer screening. False-positive recall rates, radiographic appearances, and biopsy rates were analyzed. Comparisons were made between DBT, DM, and DBT + DM, both in total and in trial year 1 compared to trial years 2 to 5, with numbers, percentages, and 95% confidence intervals (CI).

**Results:**

The false-positive recall rate was higher with DBT, 1.6% (95% CI 1.4; 1.8), compared to screening with DM, 0.8% (95% CI 0.7; 1.0). The proportion of the radiographic appearance of stellate distortion was 37.3% (91/244) with DBT, compared to 24.0% (29/121) with DM. The false-positive recall rate with DBT during trial year 1 was 2.6% (95% CI 1.8; 3.5), then stabilized at 1.5% (95% CI 1.3; 1.8) during trial years 2 to 5. The percentage of stellate distortion with DBT was 50% (19/38) trial year 1 compared to 35.0% (72/206) trial years 2 to 5.

**Conclusions:**

The higher false-positive recall rate with DBT compared to DM was mainly due to an increased detection of stellate findings. The proportion of these findings, as well as the DBT false-positive recall rate, was reduced after the first trial year.

**Clinical relevance statement:**

Assessment of false-positive recalls gives information on potential benefits and side effects in DBT screening.

**Key Points:**

• *The false-positive recall rate in a prospective digital breast tomosynthesis screening trial was higher compared to digital mammography, but still low compared to other trials*.

• *The higher false-positive recall rate with digital breast tomosynthesis was mainly due to an increased detection of stellate findings; the proportion of these findings was reduced after the first trial year*.

## Introduction

Digital breast tomosynthesis (DBT) has the potential to replace or complement digital mammography (DM) in breast cancer screening. Before implementing a new breast cancer screening method, false positives should be analyzed to further understand the consequences of the new method. The majority of recalls in breast cancer screening are false positives, and they often cause psychosocial distress and may lead to less re-attendance [1–4]. The risk of breast cancer is higher in women after a false-positive mammography screening compared to a true negative screening [5, 6]. Prospective European trials have shown both higher and lower false-positive recall rates in screening with DBT compared to DM [7–10], whereas retrospective trials from the USA have shown lower false-positive recall rates compared to DM [11].

Information about false-positive recall characteristics in screening with DBT, other than rates, is limited. A study investigating finding types with combined DBT/DM compared to DM only, leading to true-positive and false-positive examinations, found that in both groups, the mammographic appearance of asymmetry led to most false-positive examinations, followed by calcifications [12]. An analysis of the false-positive recalls in the Oslo Breast Tomosynthesis Screening Trial showed that the lower false-positive rate with DBT plus DM compared to DM was due to fewer asymmetric densities [13]. Similar results were shown in the randomized controlled To-Be trial [14]. In the first half of the Malmö Breast Tomosynthesis Screening Trial (MBTST), comparing one-view DBT to two-view DM, the false-positive rate was higher for DBT alone compared to DM alone, mainly due to a higher recall of stellate distortions. The false-positive recall rate was lower over time, indicating a learning curve [15]. Results from the whole trial are important for comparison with other trials. Also, information on false positives recalled with DBT only after an initial stabilization in the false-positive recall rate will add further insight into the early clinical experiences of DBT in screening.

In this study, we evaluated the total number of false-positive recalls including radiographic appearances and false-positive biopsies in the MBTST. We also analyzed the false-positive recall rates and appearances the first trial year compared to trial years 2 to 5.

## Materials and methods

### The Malmö Breast Tomosynthesis Screening Trial (MBTST)

The MBTST is a prospective, population-based one-arm screening trial comparing one-view DBT (mediolateral oblique view, no synthetic images) with two-view DM (craniocaudal and mediolateral oblique views) in breast cancer screening (ClinicalTrials.gov: NCT01091545). A random sample of women in the screening population in Malmö, Sweden, age 40 to 74 years old, were invited through letter between January 27, 2010, and February 13, 2015. In total 21,691, women were invited to participate. Exclusion criteria were pregnancy and non-Swedish non-English-speakers. Participating women gave written informed consent. The trial was approved by the local ethics committee at Lund University.

Participating women had one-view DBT with a wide-angle (50°) system (Mammomat Inspiration, Siemens Healthineers) and two-view DM, acquired with the same machine, at one screening occasion. In total, seven radiologists, with 2 to 41 years of breast radiology experience, were readers in the trial. The images were read independently in two separate reading groups, the DBT reading group and the DM reading group, by two radiologists in each group. The reading procedure has been described in detail elsewhere [9].

### False-positive recalls

False-positive recalls were defined as recalled women who were not diagnosed with breast cancer at screening work-up. Women with breast cancer were identified through cross-linkage with the Cancer Registry South. The false-positive recalls were divided into three separate groups based on the reading arm/s where the finding leading to recall was observed: DBT group (i.e., recalled only in the DBT reading arm), DM group (i.e., recalled only in the DM reading arm), and DBT + DM reading (i.e., recalled both in the DBT reading arm and the DM reading arm). Recalled women underwent work-up according to local routine, commonly including further imaging (typically DM and ultrasound) and if needed, fine needle aspiration and/or core needle biopsy. At the time of the trial, fine needle aspiration was still used in the routine work-up of suspicious findings at our institution.

### Variables of interest

Radiographic appearance of the dominant imaging finding leading to recall was assessed through the report from the consensus meeting or the report from the initial work-up through the Radiology Information System, divided into the following categories: stellate distortion (the finding includes distortions and a density with stellate configuration), circumscribed mass, indistinct density, architectural distortion (parenchymal disorganization without stellate configuration), focal asymmetry, calcifications, and other (such as nipple retraction or skin thickening). Self-reported breast symptoms, with no imaging findings, could also be reason for recall. If there was no clear description of the appearance in the reports, the images were retrospectively reviewed by three or more members in a panel consisting of three breast radiologists, one radiology resident, and two medical students, who categorized the appearance in consensus. At least one of the panel members was a breast radiologist. Categorization of false positives recalled in both reading groups was performed based on the appearance at DBT if there was a discrepancy between the modalities. The outcome of the work-up was divided into the following categories: normal breast tissue, benign cyst, benign calcifications, fibroadenoma, benign lesion not otherwise specified, radial scar, surgical scar tissue, and other (such as skin lesions). The outcome was primarily based on the result of biopsy, retrieved through pathology reports. If no biopsy was performed, the outcome was based on the description of the outcome in the imaging report or, if not clearly stated in the report, by the panel in consensus. Surgery was defined as any surgical procedure, such as open surgical biopsy and breast-conserving surgery.

The work-up time was defined as the time period between the screening examination and until breast cancer was ruled out, which could include one or several visits to the breast imaging unit and/or one or several visits to the breast surgery clinic. The number of imaging exams, i.e., the number of DM, ultrasound, DBT, and/or magnetic resonance exams, during work-up was retrieved through the Radiology Information System. All women were also followed until the next scheduled screening examination, i.e., 18 to 24 months.

False-positive recall rates, appearances, and outcomes were compared between the reading groups. The same parameters were also analyzed the first trial year (trial year 1) compared to trial years 2 to 5 in the DBT reading group and the DM total group (DM reading group and DBT + DM group).

Some women diagnosed with the high-risk lesion lobular carcinoma in situ go through breast surgery. They are not considered false-positive lesions in this study, but are presented for data completeness.

### Statistical analyses

Descriptive methods (numbers and percentages) were used to analyze and present data in the reading groups. The false-positive recall rate was defined as the number of false-positive recalls per 100 screened women (%) and the false-positive biopsy rate as the number of biopsies (fine needle aspiration and core needle biopsy) per 100 false-positive recalls (%) and were calculated with 95% confidence intervals (CI). The subgroups were not compared other than with numbers and percentages because of small sample sizes. Since a large proportion of women in the DBT + DM group were recalled due to symptoms and not imaging findings, the focus was on differences between the DBT group, i.e., the additional false positives, and the DM group.

## Results

**Fig. 1:**
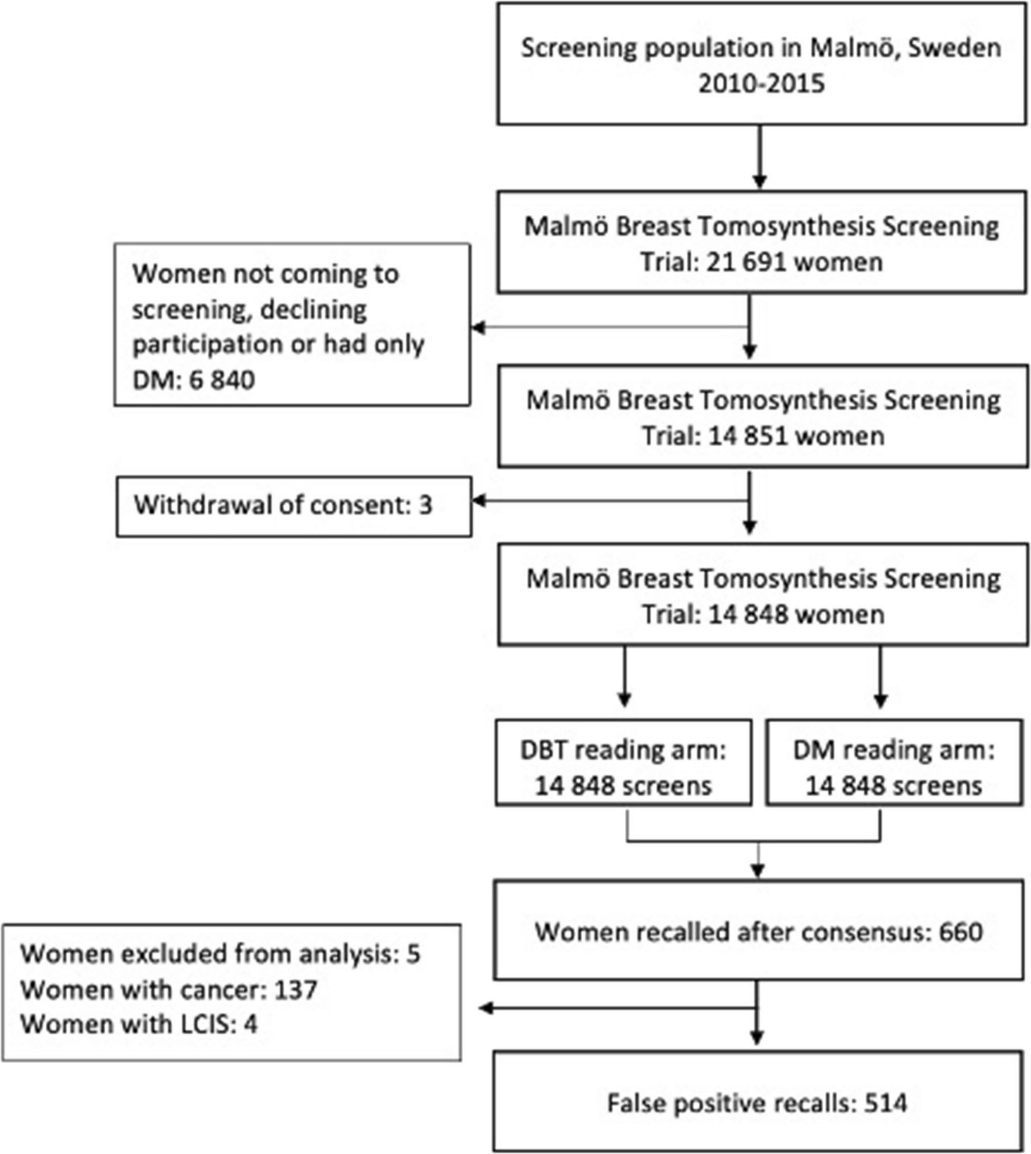
Flowchart of population and design in the Malmö Breast Tomosynthesis Screening Trial. DBT, digital breast tomosynthesis; DM, digital mammography; LCIS, lobular carcinoma in situ

**Fig. 2:**
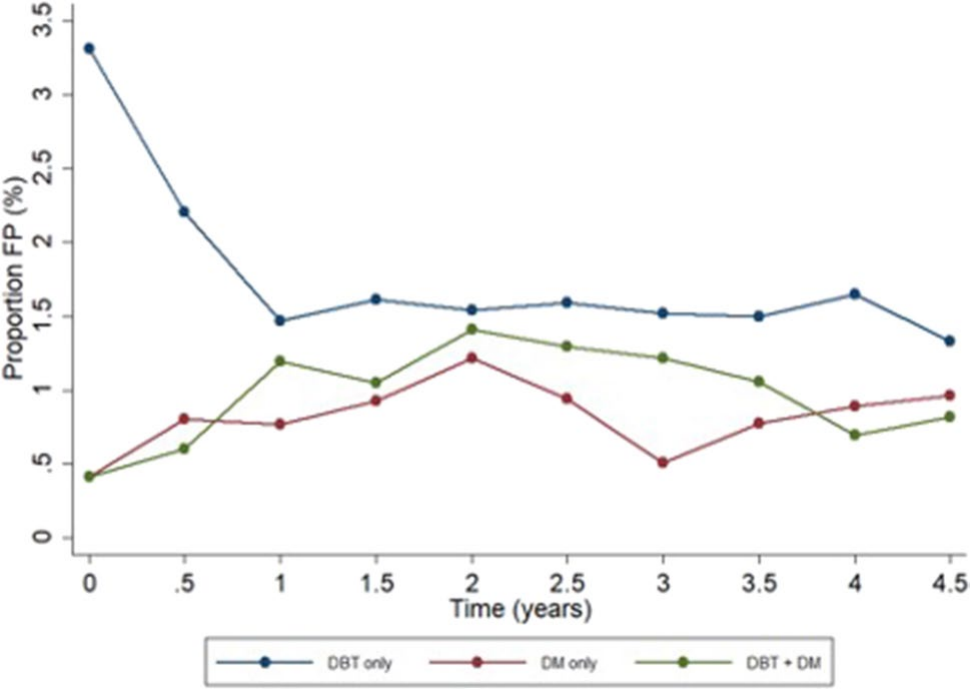
False-positive recall rate over time in the Malmö Breast Tomosynthesis Screening Trial for digital breast tomosynthesis (DBT) reading group only, digital mammography (DM) reading group only, and recalls in both reading groups (DBT + DM). FP, false-positive recalls

In total, 14,848 women participated in the MBTST and 660 women were recalled for work-up. One woman was excluded from the analysis due to lymphoma, one woman due to known distant metastases from breast cancer at screening, and three due to declining work-up. There were 137 women with breast cancer and 514 false-positive recalls (Fig. 1). Mean age at screening in women with false-positive recalls was 53 years (standard deviation ± 9.7). The false-positive recall rate was 3.5% (514/14,848, 95% CI: 3.3; 3.8) in total, 1.6% (244/14,848, 95% CI: 1.4; 1.8) in the DBT group, 0.8% (121/14,848, 95% CI: 0.7;1.0) in the DM group, and 1.0% (149/14,848, 95% CI: 0.9; 1.1) in the DBT + DM group (Table 1). The false-positive recall rate in the DBT group was higher during trial year 1, 2.6% (38/1480, 95% CI: 1.8; 3.5) and then stabilizing at 1.5% (206/13,368, 95% CI: 1.3; 1.8). The false-positive recall rate in the DM group varied between 0.5 and 1% throughout the trial (Fig. 2).

**Table 1:** False positives in the Malmö Breast Tomosynthesis Screening Trial. False-positive recalls and characteristics in different reading groups. Numbers within brackets are percentages if not otherwise stated. *DBT* digital breast tomosynthesis, *DM* digital mammography, *CI* confidence interval, *NOS* not otherwise specified

Parameters	DBT group	DM group	DBT + DM group	All
Mean age (± standard deviation)	54 ± 9.5	54 ± 10.0	52 ± 9.7	53 ± 9.7
Number of false positives	244	121	149	514
False-positive recall rate, % (95% CI)	1.6 (1.4; 1.8)	0.8 (0.7; 1.0)	1.0 (0.9; 1.1)	3.5 (3.3–3.8)
Density (BI-RADS 4^th^ Ed (18))				
BI-RADS 1 + 2 (%)	93 (38.1)	45 (37.2)	50 (33.6)	188 (36.6)
BI-RADS 3 + 4 (%)	151 (61.9)	76 (62.8)	99 (66.4)	326 (63.4)
Radiographic appearance of lesion				
Stellate distortion (%)	91 (37.3)	29 (24.0)	17 (11.4)	137 (26.7)
Circumscribed mass (%)	64 (26.2)	36 (29.8)	48 (32.2)	148 (28.8)
Calcifications (%)	19 (7.9)	11 (9.0)	15 (9.9)	45 (8.8)
Indistinct density (%)	55 (22.5)	29 (24.0)	11 (7.4)	95 (18.5)
Architectural distortion (%)	1 (0.4)	2 (1.7)	1 (0.7)	4 (0.8)
Focal asymmetry (%)	1 (0.4)	4 (3.3)	0 (0)	5 (1.0)
Retraction (%)	1 (0.4)	0 (0)	0 (0)	1 (0.2)
Edema/skin thickening (%)	3 (1.2)	1 (0.8)	0 (0)	4 (0.8)
Symptoms (%)	9 (3.7)	9 (7.4)	57 (38.3)	75 (14.6)
Outcome				
Normal breast tissue (%)	139 (57.0)	61 (50.4)	40 (26.8)	240 (46.7)
Benign cyst (%)	34 (13.9)	25 (20.7)	44 (29.5)	103 (20.0)
Benign calcifications (%)	15 (6.1)	13 (10.7)	14 (9.4)	42 (8.2)
Fibroadenoma (%)	8 (3.3)	6 (5.0)	12 (8.1)	26 (5.1)
Benign lesion NOS (%)	9 (3.7)	4 (3.3)	10 (6.7)	23 (4.5)
Radial scar (%)	12 (4.9)	0 (0)	4 (2.7)	16 (3.1)
Post-surgery (%)	10 (4.1)	1 (0.8)	2 (1.3)	13 (2.5)
Other (%)	15 (6.1)	11 (9.1)	22 (14.8)	48 (9.3)
Missing (%)	2 (0.8)	0 (0)	1 (0.7)	3 (0.6)

**Fig. 3:**
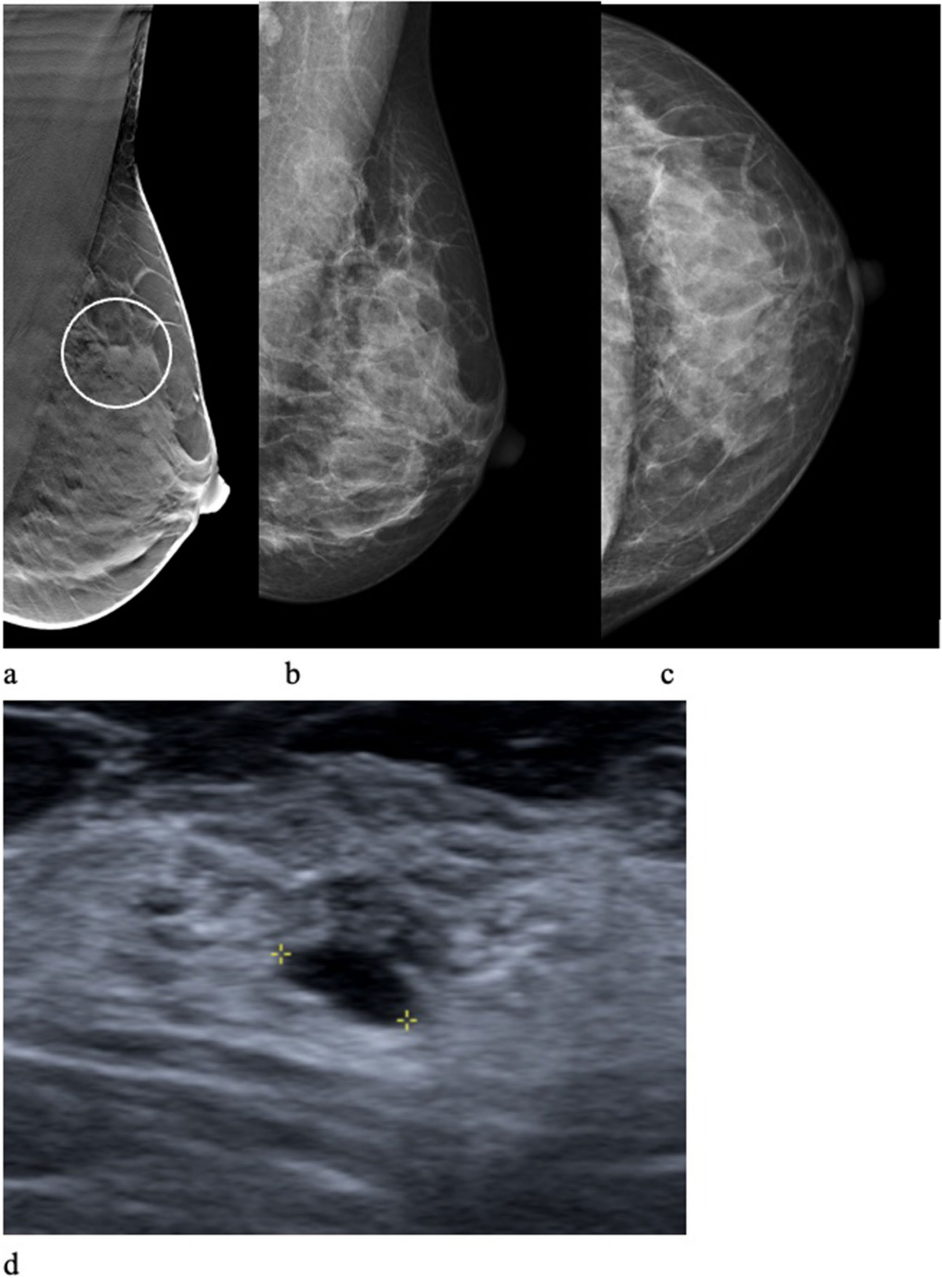
A 49-year-old woman was recalled in the DBT group due to a circumscribed mass (circle). One-view digital breast tomosynthesis (**a**) and mediolateral oblique (**b**) and craniocaudal (**c**) views at digital mammography at screening. Ultrasound (**d**) showed a benign cyst confirmed by fine needle aspiration

**Fig. 4:**
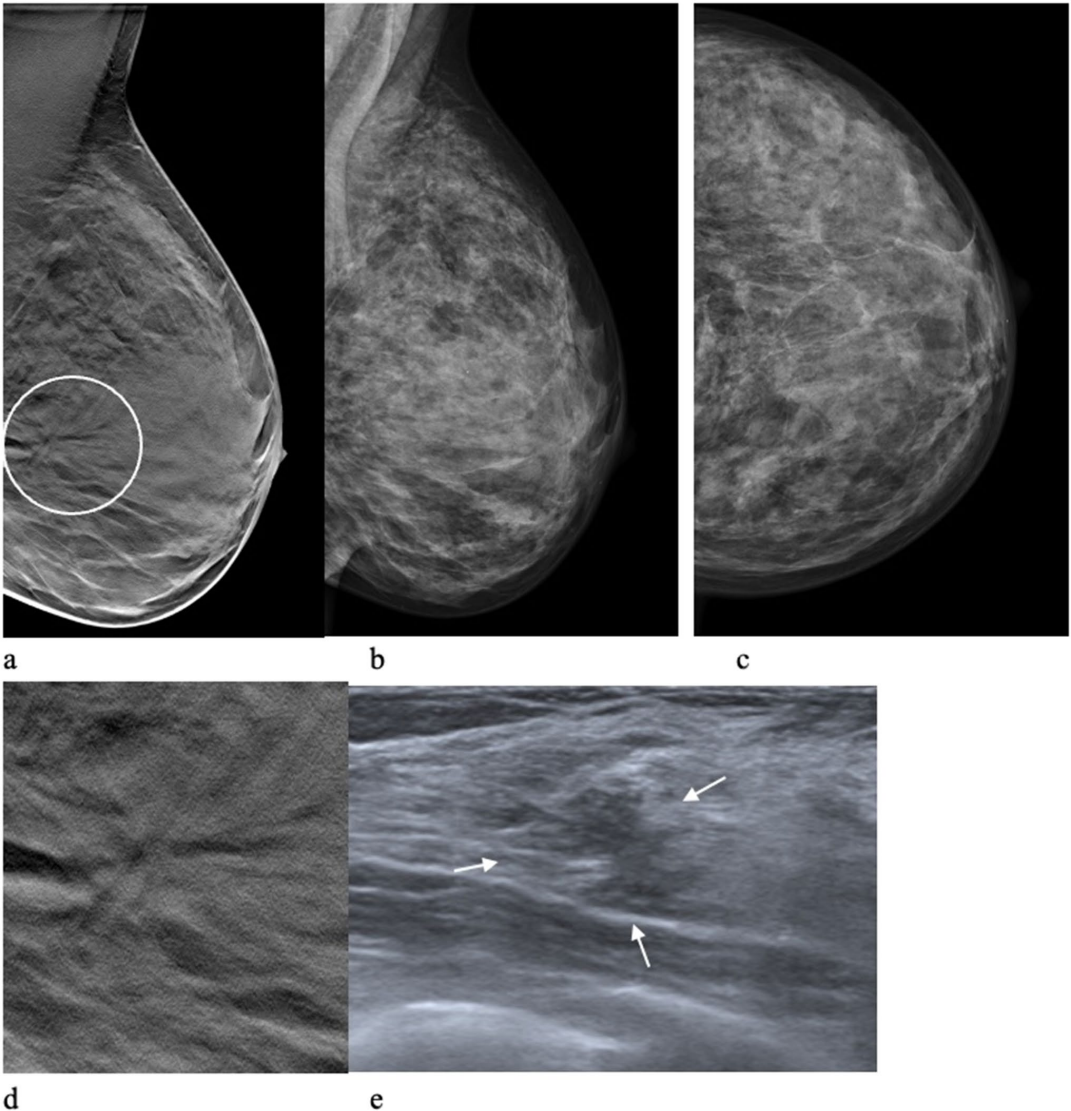
A 48-year-old woman who was recalled in the DBT group due to a stellate distortion (circle). One-view digital breast tomosynthesis (**a**) and mediolateral oblique (**b**) and craniocaudal (**c**) views at digital mammography and at screening. The false-positive finding enlarged (**d**). Ultrasound at work-up (**e**) showed a diffuse irregular lesion (arrows). Core needle biopsy confirmed a radial scar

The most common radiographic appearance of false-positive recalls in the DBT group was a stellate distortion, 37.3% (91/244), whereas the most common radiographic appearance in the DM group was a circumscribed mass, 29.8% (36/121). In the DBT + DM group, the most common reason for recall was symptoms, 38.3% (57/149). Normal breast tissue was the dominant work-up outcome in both the DBT group, 57.0% (139/244) and in the DM group, 50.4% (61/121) (Table 1). The outcome of stellate distortions showed even higher proportions of normal breast tissue, 76.9% (70/91) in the DBT group and 96.6% (28/29) in the DM group (Table 2). Two image examples of false-positive recalls are shown in Figs. 3 and 4.

**Table 2:** Outcome of false-positive recalls with the radiographic appearance of stellate distortion in different reading groups in the Malmö Breast Tomosynthesis Screening Trial. Numbers within brackets are percentages. *DBT* digital breast tomosynthesis, *DM* digital mammography

Parameters	DBT group	DM group	DBT + DM group	All
Stellate distortion	91	29	17	137
Outcome				
Normal breast tissue (%)	70 (76.9)	28 (96.6)	11 (64.7)	109 (79.6)
Benign cyst (%)	4 (4.4)	0	0	4 (2.9)
Radial scar (%)	12 (13.2)	0	4 (23.5)	16 (11.7)
Post-surgery (%)	4 (4.4)	1 (3.4)	0	5 (3.6)
Other (%)	1 (1.1)	0	2 (11.8)	3 (2.2)

The false-positive biopsy rate was similar in the DBT group, 29.5% (72/244, 95% CI: 23.9; 35.7), and the DM group, 31.4% (38/121, 95% CI: 23.3; 40.5) and higher in the DBT + DM reading group, 61.7% (92/149, 95% CI: 53.4; 69.6). The false-positive core needle biopsy rate was also similar in the DBT group, 6.1% (15/244, 95% CI: 3.5; 9.9), and the DM group, 4.1% (5/121, 95% CI: 1.4; 9.4), but higher in the DBT + DM group, 12.1% (18/149, 95% CI: 7.3; 18.4). In the DBT group, 26.4% of lesions leading to biopsy were stellate compared to no stellate distortions leading to biopsy in the DM group. The most common outcome of biopsy was a benign cyst in all three groups. There were 12 radial scars as outcome of biopsy in the DBT group, but no radial scars in the DM group. The work-up time was longer in the DBT group, median 48 days, compared to 29 and 31 days in the DM group and in the DBT + DM group, respectively. In total, 11 of the false-positive recalled women underwent surgery (Table 3).

**Table 3:** False-positive biopsies, work-up time, and imaging work-up in the Malmö Breast Tomosynthesis Screening Trial. Numbers within brackets are percentages if not otherwise stated. *DBT* digital breast tomosynthesis, *DM* digital mammography, *CI* confidence interval, *NOS* not otherwise specified

Parameters	DBT group	DM group	DBT + DM group	All
Total number of false positives	244	121	149	514
Biopsies				
Total number of biopsied women (%)	72 (29.5)	38 (31.4)	92 (61.7)	202 (39.3)
Fine needle aspiration only (%)	57 (23.3)	33 (27.3)	74 (49.7)	164 (31.9)
Core needle biopsy only (%)	6 (2.4)	4 (3.3)	10 (6.7)	20 (3.9)
Both (%)	9 (3.7)	1 (0.8)	8 (5.4)	18 (3.5)
No biopsy (%)	172 (70.5)	83 (68.6)	57 (38.3)	312 (60.7)
False-positive biopsy rate, % (95% CI)	29.5 (23.9; 35.7)	31.4 (23.3; 40.5)	61.7 (53.4; 69.6)	39.3 (35.1; 43.7)
False-positive fine needle aspiration rate, % (95% CI)	27.0 (21.6; 33.1)	28.1 (20.3; 37.0)	55.0 (46.7; 63.2)	35.4 (31.2; 39.7)
False-positive core needle biopsy rate, % (95% CI)	6.1 (3.5; 9.9)	4.1 (1.4; 9.4)	12.1 (7.3; 18.4)	7.2 (5.3; 10.0)
Surgery (%)	2 (0.8)	1 (0.8)	8 (5.4)	11 (2.1)
No surgery (%)	242 (99.2)	120 (99.2)	141 (94.6)	503 (97.9)
Lesions leading to biopsy				
Stellate distortion (%)	19 (26.4)	0 (0)	7 (7.6)	26 (12.9)
Circumscribed mass (%)	30 (41.7)	20 (52.6)	38 (41.3)	88 (43.6)
Calcifications (%)	8 (11.1)	6 (15.8)	12 (13.0)	26 (12.9)
Indistinct density (%)	6 (8.3)	6 (15.8)	3 (3.3)	15 (7.4)
Edema/skin thickening (%)	1 (1.4)	1 (2.6)	0 (0)	2 (1.0)
Symptoms (%)	8 (11.1)	5 (13.2)	32 (34.8)	45 (22.3)
Outcome of biopsy				
Normal breast tissue (%)	6 (8.3)	2 (5.3)	6 (6.5)	14 (6.9)
Benign cyst (%)	25 (34.7)	18 (47.4)	37 (40.2)	80 (39.6)
Benign calcifications (%)	6 (8.3)	6 (15.8)	11 (12.0)	23 (11.4)
Fibroadenoma (%)	6 (8.3)	5 (13.2)	12 (13.0)	23 (11.4)
Benign lesion NOS (%)	6 (8.3)	3 (7.9)	7 (7.6)	16 (7.9)
Radial scar (%)	10 (13.9)	0 (0)	4 (4.3)	14 (6.9)
Post-surgery (%)	4 (5.6)	0 (0)	2 (2.2)	6 (3.0)
Other (%)	8 (11.1)	4 (10.5)	12 (13.0)	24 (11.9)
Missing (%)	1 (1.4)	0 (0)	1 (1.1)	2 (1.0)
Median work-up time in days (range)	48 (14–913)	29 (12–1130)	31 (8–601)	38 (8–1130)
Median work-up imaging exams (range)	2.0 (1–11)	2.0 (1–8)	2.0 (1–11)	2.0 (1–11)

The most common radiographic appearance leading to a false-positive recall in the DBT group during trial year 1 was a stellate distortion, 50% (19/38). During trial years 2 to 5, the proportion of stellate distortions was lower, 35.0% (72/206). The false-positive biopsy rate in the DBT group was lower during trial year 1, 16% (6/38, 95% CI: 6; 31) than during trial years 2 to 5, 32.0% (66/206, 95% CI: 25.7; 38.9) (Table 4).

**Table 4:** False-positive recalls in the Malmö Breast Tomosynthesis Screening Trial in trial year 1 and trial years 2 to 5 in the digital breast tomosynthesis (DBT) group and in the total digital mammography (DM) group (DM group and DMT + DM group). Numbers within brackets are percentages if not otherwise stated. *CI* confidence interval, *NOS* not otherwise specified

Parameters	Trial year 1			Trial years 2–5		
	DBT group	DM total	All	DBT group	DM total	All
Number of false positives	38	18	56	206	252	458
False-positive recall rate, % (95% CI)	2.6 (1.8; 3.5)	1.2 (0.7; 1.9)	3.8 (2.9; 4.9)	1.5 (1.3; 1.8)	1.9 (1.7; 2.1)	3.4 (3.1; 3.7)
Screened women	1480	1480	1480	13368	13368	13368
Density						
BI-RADS 1 + 2 (%)	11 (29)	6 (33)	17 (30)	81 (39.6)	89 (35.3)	170 (37.1)
BI-RADS 3 + 4 (%)	27 (71)	12 (67)	39 (70)	125 (60.7)	163 (64.7)	288 (62.9)
Radiographic appearance of lesion						
Stellate distortion (%)	19 (50)	2 (11)	21 (36)	72 (35.0)	44 (17.5)	116 (25.3)
Circumscribed mass (%)	7 (18)	6 (33)	13 (23)	57 (27.7)	78 (31.0)	135 (29.5)
Calcifications (%)	3 (8)	4 (22)	7 (13)	16 (7.8)	22 (8.7)	38 (8.3)
Indistinct density (%)	7 (18)	1 (6)	8 (14)	48 (23.3)	39 (15.5)	87 (19.0)
Architectural distortion (%)	1 (3)	0	1 (2)	0	3 (1.2)	3 (0.7)
Focal asymmetry (%)	0	0	0	1 (0.5)	4 (1.6)	5 (1.1)
Retraction (%)	1 (3)	0	1 (2)	0	0	0
Edema/skin thickening (%)	0	0	0	3 (1.5)	1 (0.4)	4 (0.9)
Symptoms (%)	0	5 (28)	5 (9)	9 (4.4)	61 (24.2)	70 (15.3)
Outcome						
Normal breast tissue (%)	20 (53)	5 (28)	25 (45)	119 (57.8)	96 (38.1)	215 (46.9)
Benign cyst (%)	4 (11)	7 (39)	11 (19)	30 (14.5)	62 (24.6)	92 (20.1)
Benign calcifications (%)	3 (8)	4 (22)	7 (13)	12 (5.8)	23 (9.1)	35 (7.6)
Fibroadenoma (%)	3 (8)	1 (6)	4 (7)	5 (2.4)	17 (6.7)	22 (4.8)
Benign lesion NOS (%)	0	0	0	9 (4.4)	14 (5.6)	23 (5.0)
Radial scar (%)	1 (3)	0	1 (2)	11 (5.3)	4 (1.6)	15 (3.3)
Post-surgery (%)	3 (8)	0	3 (5)	7 (3)	3 (1.2)	10 (2.2)
Other (%)	4 (11)	0	4 (7)	11 (5.3)	33 (13.1)	44 (9.6)
Missing (%)	0	1 (5.6)	1 (1.8)	2 (1.0)	0	2 (0.4)
Biopsies						
Total number of biopsied women (%)	6 (16)	11 (61)	17 (30)	66 (32.0)	119 (47.2)	185 (40.4)
Fine needle aspiration only (%)	5 (13)	9 (50)	14 (25)	53 (25.7)	98 (38.9)	151 (33.0)
Core needle biopsy only (%)	0	1 (6)	1 (1.8)	8 (3.9)	13 (5.2)	21 (4.6)
Both (%)	1 (3)	1 (6)	2 (3.6)	5 (2.4)	8 (3.2)	13 (2.8)
No biopsy (%)	32 (84)	7 (39)	39 (70)	140 (68.0)	133 (52.8)	273 (59.6)
False-positive biopsy rate, % (95% CI)	16 (6; 31)	61 (36; 83)	30 (19; 44)	32.0 (25.7; 38.9)	47.2 (40.9; 53.6)	40.4 (35.9; 45.0)

### Lobular carcinoma in situ

There were in total four women (mean age 55 years at screening, standard deviation ± 16) with lobular carcinoma in situ detected in the MBTST; two in the DBT group and two in the DBT + DM group. Three were recalled due to stellate distortions and one was recalled due to symptoms. All four women had surgery.

## Discussion

The false-positive recall rate in the prospective population-based Malmö Breast Tomosynthesis Screening Trial (MBTST), with 14,848 participating women, was higher in screening with one-view digital breast tomosynthesis (DBT) only, 1.6%, compared to screening with digital mammography (DM) only, 0.8%. The radiographic appearance of stellate distortion was more common with DBT, 37.3%, compared with DM 24.0%. The higher false-positive recall rate in the DBT group during trial year 1, 2.6%, was stabilized at 1.5% during trial years 2 to 5, mainly due to a lower proportion of stellate distortions over time. Of lesions leading to biopsy, 26.4% were stellate in the DBT group compared to non-stellate distortion leading to biopsy in the DM group. There were 12 radial scars diagnosed with DBT and none with DM, but apart from that, the outcome of the work-up was similar in the two modalities. Our results indicate a small increase in false-positive recalls and increased detection of stellate distortions when introducing DBT in screening. The decrease in proportion of stellate distortions over time could indicate a learning curve.

In the STORM trial, comparing DM and DBT to DM only, the overall false-positive recall rate was 5.5% (395/7 292) [16]. It showed a lower false-positive recall rate with DBT + DM compared to DM only, 1.0% (73/7 292) and 2.0% (141/7 292), respectively. In the STORM-2 trial, DBT and DM, DBT and synthetic DM, and DM only were compared in screening [7]. The false-positive recall rate was significantly higher for DBT + DM, 4.0% (381/9587) and DBT + synthetic DM, 4.5% (427/9587) compared to DM alone, 3.4% (328/9587). The Oslo Tomosynthesis Screening Trial had four reading arms, where reading arm A + B represented DM (DM and DM + computer-aided detection) and reading arm C + D represented DBT (DBT and DBT + synthetic mammogram) [8]. It showed a post-consensus false-positive recall rate at 3.2% (768/24,301) in reading arms C + D and 2.1% in the A + B reading arms, hence a slightly higher false-positive recall rate in DBT screening, as in our study. The randomized controlled To-Be trial, comparing DBT + synthesized DM to standard DM, had false-positive recall rates of 2.4% (349/14,380) and 3.4% (484/14,369), respectively [14]. The false-positive recall rates in the MBTST are in general low compared to these other trials. However, the designs in all trials are different from ours, hampering the comparison of rates between studies. All these trials, including the MBTST, show results from prevalence screening rounds with no previous DBT screening examinations for comparison. A retrospective study from the USA showed a lower false-positive recall rate with DBT + DM compared to DM in the three first DBT rounds but no difference in rounds 4 and 5 [17]. The breast cancer screening strategy in the USA is however different from the European, with for example recommendations of yearly mammography screening from the age of 40 in women at average risk [18], and the results cannot be applied directly to Europe. Increased experience, in combination with access to prior DBT examinations, could decrease the false-positive recall rates in the future.

Few studies describe the radiographic appearance of false positives in DBT screening. The To-Be trial has shown that the most common radiographic appearance of false-positive recalls with DBT was asymmetry, 28.9%, which is different from our results where only 0.4% showed focal asymmetry. Spiculated masses were uncommon, only 0.6%, whereas stellate distortions were very common in our study, 36.9% [14]. Asymmetries were also most frequent in recalls with DBT in combination with DM in a retrospective study by Kim et al (75.9%) [12]. The inconsistent results between those two studies and our trial are likely to be due to different definitions of appearances, various study populations, screening ages, and screening intervals and that the examinations were performed on DBT machines from different vendors with different acquisition angles and other technical specifications. Regardless, the distribution of lesion types that radiologists will assess in DBT screening will differ from DM screening. The proportions of calcifications in DBT false-positive recalls in the mentioned DBT screening trials were however similar, around 10%, probably because the presence of calcifications is less subjective to classify than other appearances. To the best of our knowledge, no other trial has described the distribution of false-positive recalls and appearances over time, and therefore further studies are warranted to investigate how the false-positive recall rate with DBT evolves over time.

This study has limitations. The recall decision was made after a consensus meeting where all images were available. The appearances were not clearly described in the reports in a few cases and were retrospectively reviewed, meaning that the true reason for recall may differ from the one seen retrospectively. Not all findings leading to recall were biopsied which give an uncertainty of the true outcome, but we know that only one of the false-positive recalled women was diagnosed with cancer within a minimum of 2 years of follow-up. Some women were recalled due to self-reported symptoms, which reflects the screening situation. There was no access to DBT-guided biopsy at the breast imaging unit during the trial which, at least in part, could explain the longer work-up time in the DBT group and this could also have affected the biopsy rate. The results cannot necessarily be generalized to other screening settings as the MBTST was performed in a Swedish screening setting at a single-center using a wide-angle, single-vendor DBT machine.

To conclude, the false-positive recall rate in a digital breast tomosynthesis screening trial was higher compared to digital mammography, but still low compared to other digital breast tomosynthesis screening trials. The higher false-positive recall rate with digital breast tomosynthesis was mainly due to an increased recall of stellate findings; the proportion of these findings was reduced after the first trial year. Studies on false-positive recalls and false-positive appearances in subsequent screening rounds are needed to learn how access to prior digital breast tomosynthesis examinations can affect false positives in tomosynthesis screening.

## Abbreviations

CI: Confidence interval
DBT: Digital breast tomosynthesis
DM: Digital mammography
MBTST: Malmö Breast Tomosynthesis Screening Trial
NOS: Not otherwise specified

## Declarations

### Guarantor

The scientific guarantor of this publication is Sophia Zackrisson.

### Statistics and biometry

One of the authors (AR) has significant statistical expertise.

### Study subjects or cohorts overlap

Some study objects have been previously reported (1–3).

1. Lang K, Nergarden M, Andersson I, Rosso A, Zackrisson S. False positives in breast cancer screening with one-view breast tomosynthesis: an analysis of findings leading to recall, work-up and biopsy rates in the Malmo Breast Tomosynthesis Screening Trial. Eur Radiol. 2016;26(11):3899–907.

2. Rosso A, Lang K, Petersson IF, Zackrisson S. Factors affecting recall rate and false positive fraction in breast cancer screening with breast tomosynthesis—a statistical approach. Breast. 2015;24(5):680–6.

3. Zackrisson S, Lang K, Rosso A, Johnson K, Dustler M, Fornvik D, et al One-view breast tomosynthesis versus two-view mammography in the Malmo Breast Tomosynthesis Screening Trial (MBTST): a prospective, population-based, diagnostic accuracy study. Lancet Oncol. 2018;19(11):1493–503.

### Methodology

• Prospective

• Diagnostic study

• Performed at one institution
